# Decision-Tree Based Meta-Strategy Improved Accuracy of Disorder Prediction and Identified Novel Disordered Residues Inside Binding Motifs

**DOI:** 10.3390/ijms19103052

**Published:** 2018-10-07

**Authors:** Bi Zhao, Bin Xue

**Affiliations:** Department of Cell Biology, Microbiology and Molecular Biology, School of Natural Sciences and Mathematics, College of Arts and Sciences, University of South Florida, Tampa, FL 33620, USA; bizhao@mail.usf.edu

**Keywords:** meta strategy, dual threshold, significance voting, decision tree based artificial neural network, protein intrinsic disorder

## Abstract

Using computational techniques to identify intrinsically disordered residues is practical and effective in biological studies. Therefore, designing novel high-accuracy strategies is always preferable when existing strategies have a lot of room for improvement. Among many possibilities, a meta-strategy that integrates the results of multiple individual predictors has been broadly used to improve the overall performance of predictors. Nonetheless, a simple and direct integration of individual predictors may not effectively improve the performance. In this project, dual-threshold two-step significance voting and neural networks were used to integrate the predictive results of four individual predictors, including: DisEMBL, IUPred, VSL2, and ESpritz. The new meta-strategy has improved the prediction performance of intrinsically disordered residues significantly, compared to all four individual predictors and another four recently-designed predictors. The improvement was validated using five-fold cross-validation and in independent test datasets.

## 1. Introduction

Intrinsically disordered proteins (IDPs) and intrinsically disordered regions (IDRs) play critical functions in many biological processes [1,2,3,4,5,6,7]. Among all the possible molecule mechanisms for the functions of IDPs/IDRs, a major one is that IDPs/IDRs physically interact with their partners through either conformational search or induced fit [8,9,10]. Eventually, due to them having structural flexibility, IDPs/IDRs may bind to the partners with low-affinity but high-specificity [11,12,13,14,15], and thus regulate the downstream biological processes. Clearly, to characterize the dynamic process of the interaction and the mechanism of regulation, the exact locations of those intrinsically disordered amino acids (IDAAs) involved in the interaction need to be determined. However, high-accuracy experimental methods for the detection of IDPs/IDRs/IDAAs are time-consuming and cost-inefficient. Besides, high-through experimental identification of disordered residues, although having attracted a lot of attention and approaches have been widely scouted [16], is still challenging and the methods are not currently available.

Consequently, many computational tools have been developed to identify IDPs/IDRs/IDAAs and associated molecular interactions. The Protein Data Bank (PDB) [17], while being used in the majority of cases for the three-dimensional structures of biomolecules, does contain information on residues with missing coordinates. These residues are interpreted as IDAAs. Furthermore, PDB also contains the structure of molecular complexes, which frequently provides information of molecular interactions involving IDPs/IDRs. DisProt [18], which is the first database of IDPs/IDRs, not only collects IDPs/IDRs/IDAAs, but also integrates the information of molecular partners. Similarly, IDEAL [19], another database of IDPs, incorporates the interaction networks of IDPs in the database. DisBind [20], DIBS [21], and MFIB [22] are three recently developed databases for IDPs/IDRs based molecular interactions. These databases can be used to search for IDAA/IDR/IDP, or to develop computational predictors for various purposes. In fact, both PDB and DisProt are frequently used for the development of disorder predictors. In addition, PDB contains complex structures formed between a short IDR and another protein. Many of these short IDRs are known as MoRFs (Molecular Recognition Features) [23]. MoRFs are the very first type of IDRs found in molecular interaction. Based on this discovery, many MoRF related predictors have developed, such as: MoRF [24], MoRFpred [25], MFSPSSMpred [26], MoRFchibi [27], MoRFPred-plus [28], and OPAL [29], among many others. Furthermore, many other predictors have been developed for the general binding site/regions within IDPs/IDRs, e.g., ANCHOR [30], SLiMpred [31], PepBindPred [32], DISOPRED3 [33], IUPred2A/ANCHOR2 [34], etc.

All these computational tools provide information on protein intrinsic disorder for different aspects. Databases are collections of experimentally observed examples; predictors can be used to analyze novel sequences. Disorder predictors identify the location and, to some extent, the scale of flexibility of IDRs/IDAAs; binding motif predictors spot the location of binding regions; other types of predictors may provide information on various structural features and functional roles. Frequently, the outputs of disorder predictors are used as input for other predictors to improve the prediction accuracy [32,35,36,37,38,39,40]. Clearly, accurate identification of IDAAs is very important for studies associated with protein structure, intrinsic disorder, interaction, and function. Therefore, improving the prediction accuracy of protein intrinsic disorder predictors is always desirable, though also a real challenge at present time. Furthermore, improving the prediction accuracy of IDAAs has other important impacts on basic science. With more and more IDPs/IDRs being discovered, our knowledge on the actual content of protein intrinsic disorder in nature is still elusive. Part of the reason is that the accuracy of existing computational tools is still not able to meet the requirements. Therefore, developing high-accuracy predictors is still in urgent need. In addition, it could also be expected that when developing new predictors, novel computational strategies could be innovated, and thus, make a much broader impact.

In our previous studies on the development of intrinsic disorder predictors [41,42], as well as studies by many other researchers [43,44,45,46,47], meta-strategy has been demonstrated to have multiple advantages over individual predictors that adopt a single computational strategy in the prediction. One oversimplified but straightforward explanation for the success of meta-strategy is that meta-strategy is able to combine the strengths of all individual predictors, and thus improve the prediction accuracy. Nonetheless, a direct integration of multiple individual predictors may not improve the prediction accuracy significantly [48,49], however, further integration of various data pre-processing techniques will. Data pre-processing, such as angle-shift technique in protein dihedral angle prediction, was used in artificial neural network based predictor and improved the accuracy remarkably [50]. A combination of non-linear transformation and principal component analysis-based dimension reduction together with meta-strategy was used to improve the prediction accuracy of miRNAs [48]. With these proofs, it is expected that other novel techniques can also be used to improve the prediction accuracy of protein intrinsic disorder. In this project, dual-threshold value and two-step significance voting were integrated into a decision-tree based neural network to improve the prediction accuracy of IDAAs.

## 2. Results

### 2.1. Prediction Performance of Component Predictors

**Figure 1:**
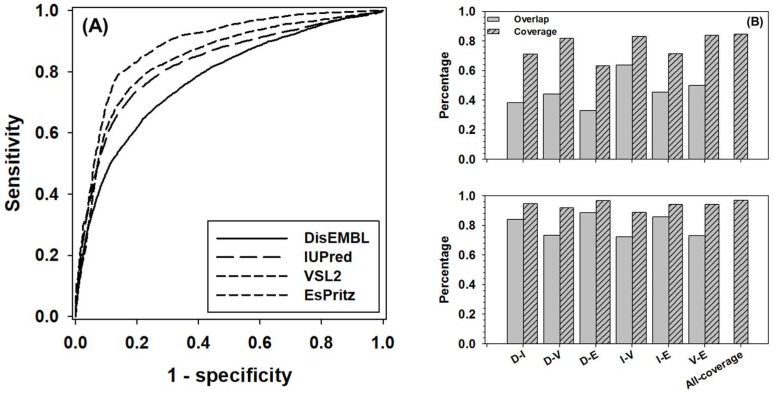
Prediction performance of four component predictors, including DisEMBL, IUPred, VSL2, and ESpritz. (**A**) ROC curves of four component predictors. The ROC curves were obtained by using the default settings of these predictors. (**B**) The pairwise overlap (gray bars) and coverage (dashed bars) for true positive predictions (upper panel) and true negative predictions (lower panel) between each pair of predictors. Axis shows pairs of predictors as follows: D-DisEMBL, I-IUPred, V-VSL2, and E-ESpritz. All-coverage on *x*-axis stands for the maximum coverage of all predictors.

The ROC (Receiver Operating Characteristic) curves of four component predictors was presented in Figure 1A. The AUC (Area Under the Curve) for DisEMBL, IUPred, VSL2, and ESpritz are 0.78, 0.82, 0.84, and 0.88, respectively. The balanced accuracy (Acc-b) of these four predictors at their default settings are: 68%, 76%, 77%, and 73%, accordingly. In Figure 1B, the overlap and coverage between every two predictors were analyzed for the positive samples (disordered residues) and negative samples (structured residues). Here, overlap stands for the ratio of true-positive (or true-negative) predictions made by both predictors over the total number of positive (or negative) samples, and coverage is defined as the ratio of correct predictions made by either predictors over the total number of samples. Clearly, the overlap of positive samples between predictors normally ranges from ~30% to 50%; however, the number for the overlap between IUpred and VSL2 went up to ~65%. In terms of coverage, the numbers were in the range from ~60% to ~80%. For negative samples, the overlap was from ~70% to ~90%, and the coverage was normally higher than ~90%. The highest values of coverage, as shown by bars at the most right-hand side of both panels, were ~85% and 97% for positive and negative samples, respectively. These two values may outline the theoretical uplimits of combining these four predictors.

### 2.2. Use Information Gain to Choose Threshold Values

**Figure 2:**
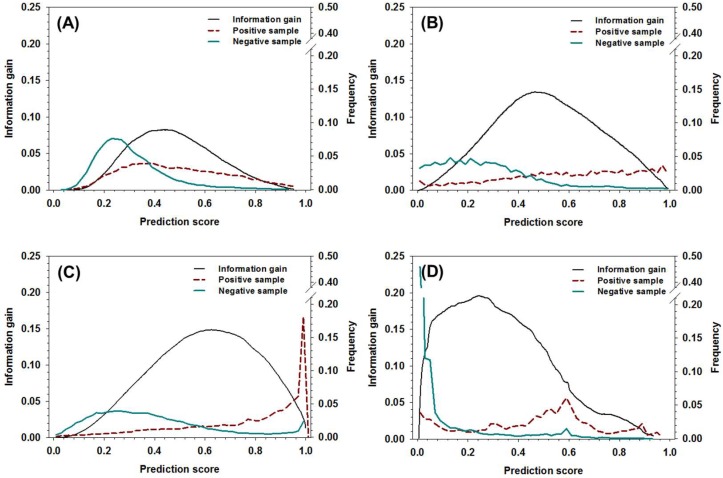
The distribution of information gain, positive sample, and negative samples as a function of prediction score for (**A**) DisEMBL, (**B**) IUPred, (**C**) VSL2, and (**D**) ESpritz. The *x*-axis shows the prediction score, the *y*-axis on the left shows the values of information gain, and the *y*-axis on the right shows the fractions of positive samples and negative samples at different prediction scores, respectively.

To use the new meta-strategy, threshold values of the decision tree need to be determined first. Other than using the method based on the distribution of positive samples and negative samples as a function of prediction score [49], the information gain of all the component predictors in the dataset was analyzed and compared to the distribution of positive samples and negative samples, as shown in Figure 2. The curves of information gain can be roughly characterized by a single-peak distribution, and the location of peaks was, roughly, on the right-hand side of the cross-point where the ratio of positive samples surpassed negative samples. More specifically, the locations of peaks for DisEMBL, IUPred, VSL2, and ESpritz were around 0.5, 0.52, 0.64, and 0.26, respectively. By notation, the locations of the peaks provide a rough estimation of the threshold values, which can be used to maximally partition positive samples and negative samples. Clearly, these values can hardly be determined by the analysis of distribution of positive and negative samples.

### 2.3. Performance of the New Predictor

Table 1 shows the prediction performance of the new meta-strategy compared to the component predictors, as well as another four recently-developed predictors under five-fold cross-validation. In brief, the performance of new meta-strategy developed in this project was obviously better than others. In terms of accuracy (Acc), balanced accuracy (Acc-b), Matthews Correlation Coefficient (MCC), F1 score, Area Under ROC Curve (AUC_ROC), and Under Precision-Recall Curve (AUC_PR), the new prediction strategy achieved 84.2%, 83.1%, 0,635, 0.744, 0.899, and 0.788, respectively, and was ranked at the first place among all eight different predictors. The new meta-strategy was ranked at the second place on sensitivity (Sens), with one percentage point behind VSL2. With regard to specificity (Spec), the new strategy was inferior to the predictors ESpritz (94%), DisEMBL (91.4%), AUCpreD (90.9%), IUPred2 (87.7%), and IUPred (87.4%). Regardless, it should be noted the Sens values of these predictors are at least 15 percentage points lower than the new meta-strategy.

The performance of all these nine predictors was also assessed using the independent dataset as shown in Table 2. By comparing the data of Table 1 and Table 2, it is obvious that although the numbers have fluctuations, the overall levels and trends of all the measures of prediction performance are essentially the same.

**Figure 3:**
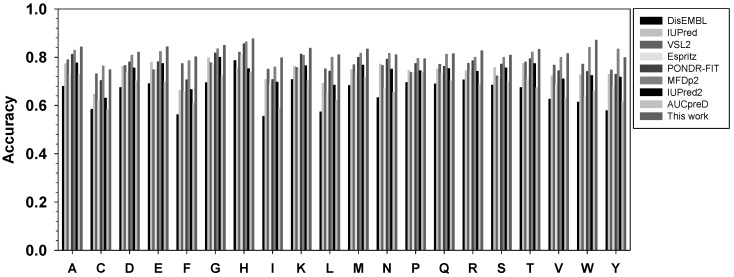
Comparison of balanced accuracy for twenty types of amino acids. The *x*-axis shows amino acid types in the alphabetic order, while the *y*-axis shows the value of balanced accuracy. For each type of amino acid, the predictors from left to right are: DisEMBL, IUPred, VSL2, ESpritz, PONDR-FIT, MFDp2, IUPred2A, and AUCpreD.

The performance of this new meta-strategy, as well as other predictors, for twenty types of amino acids was analyzed using balanced accuracy in Figure 3. Overall, the new meta-strategy has the highest Acc-b values in fifteen types of residues. The new meta-strategy was also ranked first together with the more recent predictor MFDp2 for residues P and Q. However, the new meta-strategy was ranked at the second position for C, N, and Y, with several percentage points behind MFDp2.

**Figure 4:**
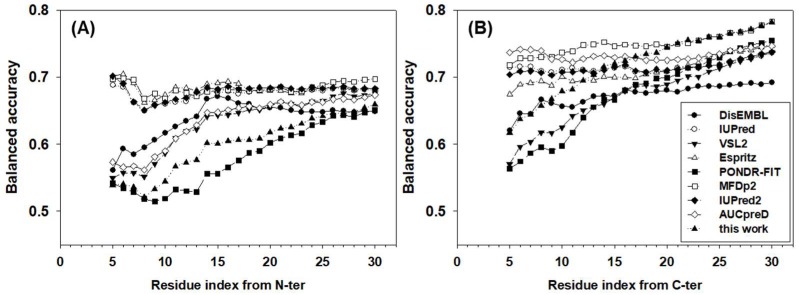
Balanced accuracy of (**A**) N-terminal and (**B**) C-terminal residues. The *x*-axis shows the distance from the first (N-terminal) or the last (C-terminal) residue. The analysis starts at the fifth residue on both N- and C-termini. The *y*-axis shows the value of the balanced accuracy.

The balanced accuracies of all predictors for terminal residues were also analyzed in Figure 4. Obviously, the accuracy is location and predictor dependent. For many predictors, the closer to the termini, the lower the accuracy. For N-terminal residues, IUPred, ESpritz, MFDp2, and IUpred2 achieved ~67% balanced accuracy, which was also largely location independent. For DisEMBL, VSL2, and AUCpreD, the balanced accuracies increased gradually from ~55% to ~65% in the window from the 5th to the ~15th residues and then kept similar accuracy afterwards. The newly designed meta-strategy had a lower balanced accuracy of ~52% for the first several residues. The accuracy then increased gradually to ~63% at the 25th residue. PONDR-FIT, a more recently developed predictor, was the least accurate predictor for N-terminal residues, especially in the range from the 10th to the 20th residues where its accuracy was 2–5 points lower than the new strategy. For C-terminal residues, the patterns of accuracy were different from N-terminal residues. First, the balanced accuracy was higher in general than N-terminal residues by several percentage points. Second, although the accuracies of predictors were still either location-independent or location-dependent, the values of accuracies were highly diversified. AUCpreD, MFDp2, IUPred, IUPred2, and ESpritz made location-independent predictions for C-terminal residues, however, the accuracy of these predictors spread from ~74% to 68%, accordingly. DisEMBL, VSL2, and PONDR-FIT’s accuracy increased gradually from ~55 to62% at the 5th residue to ~67% at the 20th residue. The accuracy of the newly designed strategy for C-terminal residues was at the lower-end for the first several terminal residues, though increased consistently and achieved the highest balanced accuracy for residues at the ~20th position.

With these observations, all the samples were regrouped into three new datasets each containing the first 25 N-terminal residues, the first 25 C-terminal residues, and the middle region, respectively. The meta-strategy was re-trained in three different datasets separately. The prediction performance of all predictors in all three regions under five-fold cross-validation was compared and analyzed in Table 3. Evidently, compared to the results in Figure 4, the prediction accuracy of terminal residues improved substantially. More specifically, the values of improvement of accuracy, balanced accuracy, F1, MCC in N-ter, Mid, and C-ter datasets ranged from 1 to 5 percentage points. For sensitivity and specificity, since many other predictors were trained to maximize either sensitivity or specificity, the new meta-strategy was normally not able to compete with them.

**Figure 5:**
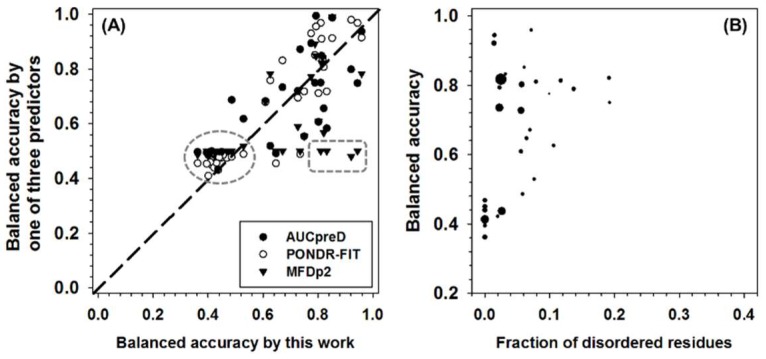
(**A**) Comparison of per-sequence balanced accuracy among AUCpreD (filled circle), PONDR-FIT (open circle), MFDp2 (filled triangle), and this work on sequences in the CASP10 test dataset. The reasons for selecting these predictors are: (1) they are developed in recent years; (2) they have higher performance on some of the accuracy measures; (3) for simplicity of visualization, only four predictors were selected. The *x*-axis shows the per-sequence balanced accuracy of this work, and the *y*-axis shows the per-sequence accuracy of the other three predictors. (**B**) Per-sequence balanced accuracy of this work (*y*-axis) as a function of the fraction of experimentally validated intrinsically disordered amino acids (IDAAs) (*x*-axis). The size of the symbol is proportional to the length of the sequence.

The performance of all the predictors were then tested in CASP10 dataset and then compared to DISOPRED3, which is one of the two best predictors in CASP10 competition (see Appendix A for more details). In brief, DISOPRED3 and AUCpreD have very similar performance and are better than other predictors on multiple measures, such as specificity, accuracy, MCC, F1, and AUC-ROC. PONDR-FIT achieved the highest balanced accuracy. The new meta-strategy has the highest sensitivity. In addition to the whole dataset analysis, the per-sequence accuracy was also analyzed. The balanced accuracy of PONDR-FIT, MFDp2, AUCpreD, and the new meta-strategy in CASP10 dataset was compared in Figure 5A. All the symbols above the diagonal line represent sequences with higher accuracy when predicted using PONDR-FIT, MFDp2, or AUCpreD, and vice versa. For symbols in the dashed circle, the prediction accuracies of the compared four predictors are all not satisfactory. Symbols in dashed box constitute another group of sequences of which the prediction accuracy of the new meta-strategy is much higher than the other three predictors. For pair-wise comparison between predictors, there are more open circles above the diagonal line, more triangles under the diagonal line, and similar numbers of filled circles on both sides of the diagonal line. Thus, PONDR-FIT (open circles) has better per-sequence prediction performance in the CASP10 dataset. The new meta-strategy and AUCpreD achieved similar results on per-sequence prediction performance. Since the new meta-strategy also made a very low-accuracy prediction on some of the sequences, analyzing the potential reasons could be beneficial. For this purpose, the per-sequence balanced accuracy, fraction of experimentally validated IDAAs per sequence, and the length of each sequence were analyzed in Figure 5B. In this figure, it is apparent that sequences with a very low fraction of experimentally validated IDAAs have very low accuracy. Therefore, the fraction of IDAAs is a critical factor for the performance of the new meta-strategy.

## 3. Discussion

Intrinsically disordered proteins play critical roles in biomolecular interaction and signaling; therefore, identifying these residues is crucial for the subsequent analysis and biological studies of the functions and mechanisms. Many experimental techniques have been designed for characterizing these residues. Nonetheless, these techniques are normally time-consuming and/or cost-inefficient. Besides, these techniques may not be appropriate for proteomic studies, although many new approaches are under development [16,51,52]. Therefore, using computational tools to predict intrinsically disordered residues becomes practical, especially for novel protein sequences. Under this situation, using high-accuracy predictors is essential. However, as shown in the previous analysis, the current levels of prediction accuracy of many disordered predictors still have a lot of room for improvement.

There are multiple ways to improve the accuracy of machine learning based techniques. Tuning the list of input features is often the first trial. Recently, deep learning and meta-strategy have also been applied to improve the prediction accuracy. Our previous studies and the studies of other groups [41,42,43,44,45,46,47] a direct application of meta-strategy may not lead to the improvement of prediction accuracy, although it has been demonstrated that meta-strategy has many advantages [48]. In these cases, novel data processing techniques are very helpful [48,49]. Therefore, in this project, a dual-threshold was employed; two-step voting with different accuracy stringency was also integrated in the pipeline, based on the analysis of information gain. These techniques eventually contributed remarkably to the improvement of prediction accuracy. The outcomes of this new strategy demonstrate that: (1) integrating lower-accuracy predictors is able to produce higher-accuracy output; (2) the improvement of prediction performance of meta-strategy is significant and impressive, compared to individual predictors and other state-of-the-art predictors, including deep-learning based predictors; (3) the meta-strategy has well-balanced results for sensitivity and specificity, and therefore, is able to achieve higher values on other evaluation quantities, such as F1, MCC, etc.; (4) the meta-strategy provides novel ideas on the renovation of existing predictors.

Many data-processing techniques could be integrated into the meta-strategy. In this project, dual-threshold and two-step significance voting were designed and were critical for the improvement of prediction performance. Dual-threshold refers to true prediction and false prediction having different threshold values. By using dual-threshold, it is possible to control the increase of false positive rate and false negative rate. Two-step voting is a technique to use two sets of threshold values at two steps. At the first step, a set of more stringent threshold values are used, and at the second step less-stringent threshold values are used. In this way, the results from the first step have higher reliability than the second step. Significance-voting is another very useful technique complementary to the well-known majority-voting. When using majority-voting, the number of predictors making true predictions and the number of predictors making false predictions competes to determine the final results. In the application of significance-voting, the Euclidean distance of a prediction score from the corresponding threshold value is calculated, then the sum of distances of predictors making true predictions is compared to that of predictors making false predictions. Clearly, this technique is also beneficial for reducing the prediction error. For majority-voting based strategy, overlap is a critical measurement. However, in significance-voting based predictor, although overlap is still very important, coverage plays a more critical role. In addition, results from majority-voting and from significance-voting predictors have different preferences. Majority-voting is strong in selecting part of the true predictions that have very high confidence. However, significance-voting is able to pick up additional true predictions that cannot be identified by majority-voting.

When selecting individual predictors, overlap and coverage between a pair of predictors or among multiple predictors can be calculated and used to check the similarity of two predictors, and to evaluate whether the combination of these two predictors is able to improve final prediction accuracy. If the two predictors have extremely high overlap and very low coverage, these two predictors are very similar to each other in terms of the predictive results, and vice versa. Evidently, these two types of situations need to be avoided in most cases when selecting the component predictors. Normally, the selected component predictors should have a reasonably level of overlap and a higher level of coverage. The values of coverage also provide an estimation on the maximum values of true-positive and true-negative predictions by combining a pair or several predictors.

It should also be noted that most experimental work aiming at IDAA validation is focused on in vitro approaches, and consequently, the corresponding data analysis and computational strategies are also focused on in vitro data. Regardless, the in vitro foldability of amino acid residues could be very different from in vivo environment [53]. Therefore, novel ideas to develop large-scale in vivo conformational assays are also urgently needed. In fact, novel in vivo labeling strategies of IDAAs have been proposed [53]. It is hopeful that these in vivo techniques or at least the data of in vivo studies will be eventually incorporated into novel predictors of in vivo foldability of IDAA.

## 4. Materials and Methods

DisProt v7.0 and PDB (Protein DataBank) were combined to build the dataset of disordered residues. DisProt contains over 800 protein sequences, in which the IDAAs/IDRs have been identified using various experimental techniques, such as X-ray, NMR, circular dichroism (CD) spectrometry, proteolysis, etc. For all the DisProt sequences, IDAAs have already been annotated. PDB sequences were extracted using the PISCES server [54]. All the PDB structures in the list have 2.5 angstrom or better resolution and 30% or less sequence identity. Then, 20% of the PDB sequences were randomly selected for further analysis. The missing residues in these PDB sequences were assigned as IDAAs, while all other residues were determined to be structured residues. All the extracted sequences from both DisProt and PDB were further filtered using CD-HIT [55] to remove sequences with 30% or higher sequence identity. Finally, there are 312 protein sequences, containing 30,140 disordered residues and 75,945 structured residues. All the sequences with X-ray structures in CASP10 [56] were also extracted. These sequences were each aligned with all the sequences in the above-mentioned main dataset to check the sequence identity. Only sequences with 30% or lower sequences identity were kept to make the second independent test dataset. This second independent test dataset has 35 sequences.

**Figure 6:**
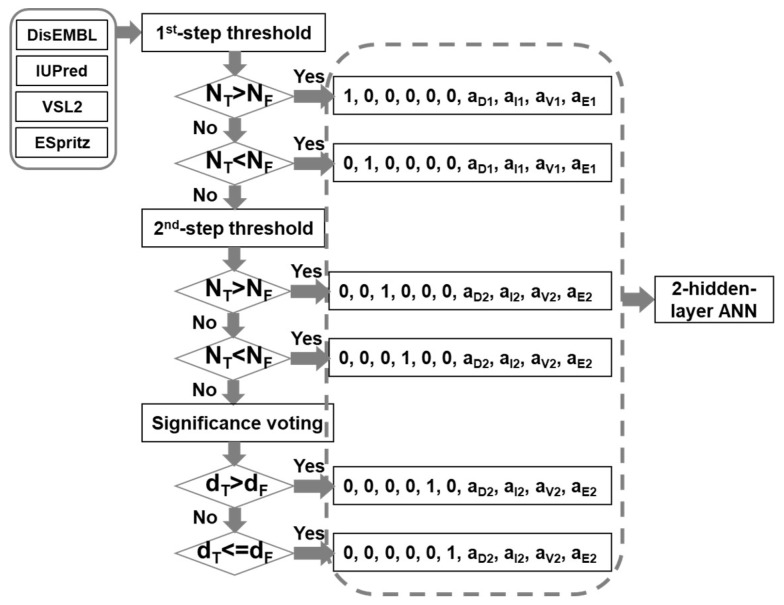
Infrastructure of the new meta-strategy. N_T_ and N_F_ are the numbers of predictors making true prediction and false prediction, respectively. “a1” and “a2” are the differences of prediction score from the 1st-step threshold and the 2nd-step threshold values, respectively. The letter subscripts represent DisEMBL (D), IUPred(I), VSL2(V), and ESpritz(E), accordingly. “d_T_” and “d_F_” are Euclidean distances of prediction scores from their corresponding threshold values for true predictions and false predictions, accordingly.

The infrastructure of the meta-strategy is shown in Figure 6. The prediction results of DisEMBL [57], IUPred [58], VSL2 [59], and ESpritz [60], were used as input. The major reasons for choosing these four predictors are as follows: (1) these predictors were designed using very different strategies. DisEMBL uses artificial neural networks. IUPred uses knowledge-based interaction potential. VSL2 uses neural networks on sequences of different lengths. ESpritz applied bidirectional recursive neural network (BRNN) and was trained separately on N-terminal, C-terminal, and the general sequences; (2) they achieved relatively higher prediction accuracy; (3) these predictors have standalone versions. These four scores were then fed into a decision-tree based artificial neural network (DBann) to make the final prediction. The DBann combines four specific techniques including dual-threshold, significance-voting, two-step selection [49], and two-hidden-layer Artificial Neural Network (ANN). Dual-threshold is a technique using different threshold values for true prediction and false predictions. Significance-voting is complementary to majority-voting by calculating the Euclidean distance of prediction scores to their corresponding threshold values and then comparing the distances of true predictions and false predictions to make selections. For example, when two predictors make true predictions and another two predictors make false predictions, comparing the number of true predictions (N_T_) and the number of false predictions (N_P_) may have limited usage. In this case, comparing the sum of distances from true thresholds value (d_T_) and the total distance from false threshold values (d_F_) provides more useful information of the relative significance of true predictions and false predictions. Two-step selection uses two sets of dual-threshold values together with significance-voting as follows: (1) use more stringent values as the first-step threshold values for both true predictions and false predictions; (2) select less stringent values as the second-step threshold values for both true predictions and false predictions; (3) if the numbers of predictors for true prediction and false prediction are equal in the first-step, second-step examination will be performed. If the numbers are still the same, the significance voting will be carried out; (4) based on the results of the above-mentioned comparison, the predictive results of individual predictors will be encoded differently. The encoded predictive results will then be fed into the two-hidden-layer ANN, which is a fully connected ANN and has ten and two nodes in the input and output layers, respectively, as well as twenty nodes in both hidden layers. The activation function for all the nodes is hyperbolic tangent function. In addition, in the output layer, the output was further transformed using a soft matrix function.

All the selected sequences were grouped into two datasets. One contains a randomly-selected 20% of all the samples and was set as the independent test dataset, while the other, containing the rest 80% of the samples, was designated as the training and validation dataset. The ratios of positive samples (disordered residues) to negative samples (structured residues) in two datasets are roughly the same. The training and validation dataset was further split into five subsets for five-fold cross-validation. In brief, three out of five subsets were used to train the predictor, the forth subset was used to prevent overfitting, and the last one was used to validate the final prediction performance. By using the different subsets for training, preventing overfitting, and validation, the aforementioned process was repeated five times. The final prediction performance was the average of all five times in the validation subsets. The trained predictors were also evaluated in the independent test dataset.

The performance of predictors was assessed using Sensitivity (Sens), Specificity (Spec), Accuracy (Acc), balanced accuracy (Acc-b, the average of sensitivity and specificity), F1 score (F1), Matthews Correlation Coefficient (MCC), Area Under ROC Curve (AUC, or AUC_ROC), and Area Under precision-recall Curve (AUC_PR) under five-fold cross-validation and in independent datasets. The performance of newly designed predictor was compared to four component predictors (DisEMBL, IUPred, VSL2, and ESpritz), as well as another four recently developed predictors, including PONDR-FIT [42], MFDp2 [61], IUPred2A [34], and AUCpreD [62].

Information Gain (IG) was calculated as a function of predictive score as follows:

(1) $$IG\left(x\right)=\underset{i=1,2}{\sum }p_{i}\log_{2}p_{i}-\underset{j=1,2}{\sum }f_{j}\left(x\right)\underset{k=1,2}{\sum }p_{j,k}\log_{2}p_{j,k}$$

In which, *p_i_* is the fraction of positive (*i* = 1) or negative (*i* = 2) samples in the dataset; “*x*” is the threshold prediction score to split the dataset into two groups; f_j_(*x*) is the fraction of samples with prediction score higher than the threshold (*j* = 1) or the fraction of samples with prediction score lower than the threshold (*j* = 2); and *p_j_*_,*k*_ refers to the fraction of positive samples (*k* = 1) or negative samples (*k* = 2) in the j-th group.

## Figures and Tables

**Table 1:** Prediction performance of the new strategy under five-fold cross-validation, in comparison with four component predictors, another four recently-designed predictors.

	DisEMBL	IUPred	VSL2	Espritz	PONDR-FIT	MFDp2	IUPred2	AUCpreD	This Work
Sens	0.440 ± 0.008	0.650 ± 0.003	**0.817 ± 0.004**	0.514 ± 0.009	0.713 ± 0.004	0.777 ± 0.004	0.640 ± 0.003	0.592 ± 0.006	0.807 ± 0.012
Spec	0.914± 0.002	0.874 ± 0.004	0.736 ± 0.003	**0.939 ± 0.002**	0.859 ± 0.004	0.859 ± 0.004	0.877 ± 0.004	0.909 ± 0.002	0.856 ± 0.007
Acc	0.779 ± 0.003	0.810 ± 0.003	0.759 ± 0.002	0.818 ± 0.004	0.817 ± 0.004	0.836 ± 0.003	0.810 ± 0.003	0.819 ± 0.003	**0.842 ± 0.003**
Acc-b	0.677 ± 0.006	0.762 ± 0.002	0.776 ± 0.002	0.726 ± 0.004	0.786 ± 0.003	0.818 ± 0.003	0.759 ± 0.002	0.751 ± 0.003	**0.831 ± 0.004**
MCC	0.410 ± 0.007	0.529 ± 0.006	0.504 ± 0.004	0.521 ± 0.007	0.561 ± 0.007	0.614 ± 0.006	0.526 ± 0.006	0.535 ± 0.006	**0.635 ± 0.006**
F1	0.531 ± 0.006	0.660 ± 0.003	0.658 ± 0.003	0.616 ± 0.006	0.689 ± 0.004	0.729 ± 0.003	0.657 ± 0.004	0.651 ± 0.005	**0.744 ± 0.004**
AUC_ROC	0.776 ± 0.004	0.823 ± 0.001	0.841 ± 0.003	0.886 ± 0.003	0.857 ± 0.003	0.879 ± 0.002	0.822 ± 0.001	0.869 ± 0.003	**0.899 ± 0.004**
AUC_PR	0.607 ± 0.007	0.675 ± 0.007	0.656 ± 0.020	0.752 ± 0.006	0.696 ± 0.004	0.629 ± 0.006	0.657 ± 0.004	0.716 ± 0.007	**0.788 ± 0.010**

Note Bene. The measures of predictor performance include: sensitivity (Sens), specificity (Spec), accuracy (Acc), balanced accuracy (Acc-b), Matthews Correlation Coefficient (MCC), F1 score, Area Under ROC Curve (AUC_ROC), and Area Under Precision-Recall Curve (AUC_PR). The highest value in each of these measures is in bold and highlighted (red).

**Table 2:** Prediction performance of all nine predictors in the independent dataset.

	DisEMBL	IUPred	VSL2	Espritz	PONDR-FIT	MFDp2	IUPred2	AUCpreD	This Work
Sens	0.454	0.656	**0.82**	0.529	0.728	0.78	0.647	0.609	0.811 ± 0.007
Spec	0.915	0.872	0.735	**0.932**	0.856	0.857	0.87	0.908	0.856 ± 0.006
Acc	0.784	0.811	0.759	0.818	0.82	0.835	0.811	0.823	**0.844 ± 0.003**
Acc-b	0.684	0.764	0.777	0.731	0.792	0.819	0.761	0.759	**0.834 ± 0.001**
MCC	0.424	0.532	0.507	0.521	0.569	0.615	0.53	0.53	**0.639 ± 0.003**
F1	0.544	0.663	0.659	0.622	0.696	0.729	0.66	0.66	**0.747 ± 0.002**
AUC_ROC	0.779	0.824	0.841	0.888	0.857	0.88	0.822	0.872	**0.9 ± 0.002**
AUC_PR	0.617	0.673	0.642	0.754	0.695	0.622	0.672	0.72	**0.789 ± 0.005**

Note Bene. The new strategy was optimized five times under five-fold cross-validation. Therefore, the performance was also tested in the independent test dataset five times. The results shown in the table is the average of all five times. The highest value in each of these measures is in bold and highlighted (red).

**Table 3:** Comparison of prediction performance under five-fold cross-validation of eight predictors, as well as the new strategy trained for N-terminal, middle region, and C-terminal residues. The highest value in each of these measures is in bold and highlighted (red).

		DisEMBL	IUPred	VSL2	Espritz	PONDR-FIT	MFDp2	IUPred2	AUCpreD	This Work
N-ter	Sens	0.553 ± 0.009	0.541 ± 0.017	0.782 ± 0.011	0.582 ± 0.010	**0.837 ± 0.004**	0.782 ± 0.009	0.539 ± 0.015	0.748 ± 0.011	0.829 ± 0.023
	Spec	0.741 ± 0.016	0.841 ± 0.020	0.524 ± 0.024	0.789 ± 0.012	0.405 ± 0.020	0.582 ± 0.039	**0.842 ± 0.020**	0.590 ± 0.038	0.572 ± 0.049
	Acc	0.614 ± 0.003	0.639 ± 0.012	0.698 ± 0.005	0.650 ± 0.004	0.697 ± 0.006	0.718 ± 0.012	0.638 ± 0.014	0.697 ± 0.011	**0.746 ± 0.014**
	Acc-b	0.647 ± 0.004	0.691 ± 0.011	0.653 ± 0.010	0.686 ± 0.004	0.621 ± 0.011	0.682 ± 0.020	0.691 ± 0.014	0.669 ± 0.016	**0.701 ± 0.020**
	MCC	0.277 ± 0.009	0.364 ± 0.021	0.308 ± 0.019	0.349 ± 0.009	0.265 ± 0.024	0.361 ± 0.038	0.363 ± 0.027	0.330 ± 0.029	**0.410 ± 0.035**
	F1	0.660 ± 0.007	0.669 ± 0.015	0.778 ± 0.006	0.692 ± 0.008	0.789 ± 0.007	0.789 ± 0.007	0.668 ± 0.014	0.770 ± 0.010	**0.815 ± 0.013**
Middle	Sens	0.387 ± 0.009	0.682 ± 0.005	0.820 ± 0.004	0.481 ± 0.010	**0.663 ± 0.004**	0.777 ± 0.005	0.672 ± 0.005	0.539 ± 0.007	0.807 ± 0.013
	Spec	0.927 ± 0.001	0.877 ± 0.004	0.751 ± 0.004	0.948 ± 0.002	0.888 ± 0.004	0.875 ± 0.005	**0.880 ± 0.004**	0.927 ± 0.001	0.877 ± 0.006
	Acc	0.801 ± 0.004	0.831 ± 0.004	0.767 ± 0.003	0.839 ± 0.004	0.835 ± 0.004	0.852 ± 0.005	0.831 ± 0.004	0.836 ± 0.003	**0.861 ± 0.004**
	Acc-b	0.657 ± 0.005	0.780 ± 0.003	0.786 ± 0.003	0.715 ± 0.005	0.776 ± 0.003	0.826 ± 0.004	0.776 ± 0.003	0.732 ± 0.003	**0.842 ± 0.005**
	MCC	0.376 ± 0.009	0.544 ± 0.005	0.497 ± 0.006	0.506 ± 0.008	0.546 ± 0.006	0.616 ± 0.008	0.540 ± 0.007	0.510 ± 0.006	**0.643 ± 0.008**
	F1	0.477 ± 0.007	0.628 ± 0.003	0.622 ± 0.005	0.583 ± 0.008	0.653 ± 0.004	0.711 ± 0.004	0.650 ± 0.004	0.606 ± 0.005	**0.731 ± 0.006**
C-ter	Sens	0.584 ± 0.014	0.615 ± 0.017	**0.847 ± 0.016**	0.609 ± 0.019	0.828 ± 0.017	0.771 ± 0.016	0.598 ± 0.016	0.681 ± 0.013	0.790 ± 0.018
	Spec	0.787 ± 0.021	0.838 ± 0.023	0.586 ± 0.014	0.857 ± 0.015	0.615 ± 0.017	0.743 ± 0.015	**0.845 ± 0.019**	0.796 ± 0.019	0.769 ± 0.021
	Acc	0.686 ± 0.013	0.727 ± 0.005	0.715 ± 0.007	0.734 ± 0.009	0.720 ± 0.008	0.757 ± 0.007	0.723 ± 0.007	0.739 ± 0.009	**0.780 ± 0.009**
	Acc-b	0.685 ± 0.012	0.726 ± 0.006	0.716 ± 0.007	0.733 ± 0.007	0.721 ± 0.009	0.757 ± 0.007	0.722 ± 0.008	0.739 ± 0.008	**0.780 ± 0.009**
	MCC	0.379 ± 0.026	0.465 ± 0.014	0.448 ± 0.015	0.482 ± 0.015	0.453 ± 0.018	0.514 ± 0.014	0.459 ± 0.018	0.481 ± 0.018	**0.560 ± 0.018**
	F1	0.649 ± 0.012	0.691 ± 0.008	0.747 ± 0.008	0.694 ± 0.009	0.746 ± 0.008	0.759 ± 0.006	0.682 ± 0.012	0.722 ± 0.007	**0.781 ± 0.006**

## Abbreviations

IDP	Intrinsically disordered protein
IDR	Intrinsically disordered region
IDAA	Intrinsically disordered amino acid
ANN	Artificial neural network
IG	Information gain
Sens	Sensitivity
Spec	Specificity
Acc	Accuracy
Acc-b	Balanced accuracy
MCC	Mathew’s correlation coefficient
AUC-ROC	Area under ROC curve
AUC-PR	Area under precision-recall curve

## Appendix A

**Table A1:** Comparison of prediction accuracy of in the CASP10 test dataset.

	Sen	Spec	Acc	Acc-b	MCC	F1	AUC_ROC	AUC_PR
Disembl	0.379	0.954	0.929	0.666	0.286	0.318	0.754	0.295
IUPred	0.168	0.958	0.924	0.563	0.122	0.161	0.618	0.175
VSL2	0.612	0.811	0.803	0.712	0.214	0.213	0.774	0.275
Espritz	0.512	0.921	0.903	0.716	0.298	0.316	0.815	0.404
DISOPRED3	0.362	**0.993**	**0.966**	0.678	**0.495**	**0.481**	**0.860**	**0.495**
PONDRFIT	0.586	0.929	0.914	**0.758**	0.362	0.374	0.830	0.358
MFDp2	0.325	0.975	0.947	0.650	0.322	0.349	0.778	0.352
IUPred2	0.164	0.959	0.924	0.561	0.119	0.158	0.616	0.170
AUCpreD	0.425	0.984	**0.960**	0.705	0.465	**0.481**	**0.863**	**0.501**
This work	**0.629**	0.840	0.831	0.734	0.249	0.245	0.793	0.305

Note Bene. DISOPRED3 is one of the two best predictors in CASP10 competition and therefore was used in the comparison. The other one and its web server were not available when the study was carried out. The highest value in each of these measures is in bold and highlighted (red).
